# Effects of population density and environmental conditions on life‐history prevalence in a migratory fish

**DOI:** 10.1002/ece3.10087

**Published:** 2023-05-23

**Authors:** Mark H. Sorel, Andrew R. Murdoch, Richard W. Zabel, Cory M. Kamphaus, Eric R. Buhle, Mark D. Scheuerell, Sarah J. Converse

**Affiliations:** ^1^ Washington Cooperative Fish and Wildlife Research Unit, School of Aquatic and Fishery Sciences University of Washington Seattle Washington USA; ^2^ Washington Department of Fish and Wildlife Olympia Washington USA; ^3^ Northwest Fisheries Science Center, National Marine Fisheries Service National Oceanic and Atmospheric Association Seattle Washington USA; ^4^ Yakama Nation Fisheries Toppenish Washington USA; ^5^ Mt. Hood Environmental Sandy Oregon USA; ^6^ U.S. Geological Survey, Washington Cooperative Fish and Wildlife Research Unit, School of Aquatic and Fishery Sciences University of Washington Seattle Washington USA; ^7^ U.S. Geological Survey, Washington Cooperative Fish and Wildlife Research Unit, School of Environmental and Forest Sciences & School of Aquatic and Fishery Sciences University of Washington Seattle Washington USA

**Keywords:** Chinook salmon, density dependence, dispersal, habitat use, individual heterogeneity, life‐history diversity, migration, reproduction

## Abstract

Individual variation in life‐history traits can have important implications for the ability of populations to respond to environmental variability and change. In migratory animals, flexibility in the timing of life‐history events, such as juvenile emigration from natal areas, can influence the effects of population density and environmental conditions on habitat use and population dynamics. We evaluated the functional relationships between population density and environmental covariates and the abundance of juveniles expressing different life‐history pathways in a migratory fish, Chinook salmon (*Oncorhynchus tshawytscha*), in the Wenatchee River basin in Washington State, USA. We found that the abundance of younger emigrants from natal streams was best described by an accelerating or near‐linear function of spawners, whereas the abundance of older emigrants was best described by a decelerating function of spawners. This supports the hypothesis that emigration timing varies in response to density in natal areas, with younger‐emigrating life‐history pathways comprising a larger proportion of emigrants when densities of conspecifics are high. We also observed positive relationships between winter stream discharge and abundance of younger emigrants, supporting the hypothesis that habitat conditions can also influence the prevalence of different life‐history pathways. Our results suggest that early emigration, and a resultant increase in the use of downstream rearing habitats, may increase at higher population densities and with greater winter precipitation. Winter precipitation is projected to increase in this system due to climate warming. Characterizing relationships between life‐history prevalence and environmental conditions may improve our understanding of species habitat requirements and is a first step in understanding the dynamics of species with diverse life‐history strategies. As environmental conditions change—due to climate change, management, or other factors—resultant life‐history changes are likely to have important demographic implications that will be challenging to predict when life‐history diversity is not accounted for in population models.

## Graphical abstract

Quantifying the relationship between spawner density, environmental conditions, and the production of juveniles expressing different life history pathways is a necessary first step in understanding drivers of population productivity. For a population of endangered Chinook salmon, we quantified how the prevalence of juveniles that emigrate from their natal stream at younger ages increased with increasing population density and winter streamflow.

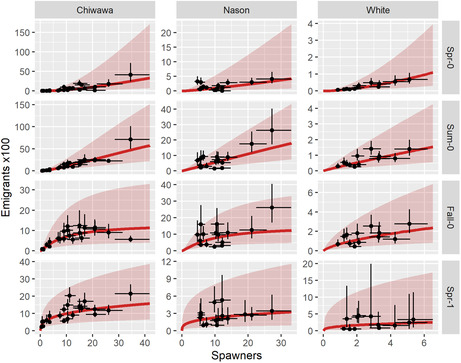

## 1 INTRODUCTION

Genetic, ontogenetic, and behavioral factors interact to shape the expression of life‐history traits, such as the timing of breeding and migration (Stearns, 1976). Within a population, variation in life‐history traits may allow individuals to exploit different niche spaces, thus reducing resource competition and expanding the total niche space exploited by a population (Raffard et al., 2019). This expansion can render populations more capable of coping with environmental variability and change (Conner & White, 1999). Furthermore, variability in life‐history traits leads to variability in demographic responses to fluctuating environmental conditions, which dampens the variability in populations over time (Schindler et al., 2010).

In migratory animals, individual variability in migratory behavior can mediate the effects of population density and environmental conditions on survival and habitat use (Shaw, 2020). Individuals may exhibit variable timing and destination of migration (Brown, van Loon, et al., 2021) and some individuals may not migrate at all (Martin et al., 2022). For example, variation in the migration timing of individual white storks (*Ciconia ciconia*) affects conditions experienced during migration and ultimately energy expenditure and wintering destination (Acácio et al., 2022). Plasticity and individual heterogeneity in migration behavior is exhibited in most migratory ungulate populations and is driven by biotic interactions, climate, and anthropogenic factors (Xu et al., 2021). In the face of substantial environmental change, diversity and flexibility in migratory behavior may have important implications for species habitat use and viability (Senner et al., 2020; Sturrock et al., 2020).

Anadromous fish migrate between freshwater spawning habitat and saltwater habitat. These species exhibit considerable diversity in life‐history traits as related to the age and extent of downstream and upstream migrations throughout their lives (Bourret et al., 2016; Thorpe et al., 1998). Multiple juvenile life‐history pathways (LHPs), defined by use of different freshwater‐rearing habitats (e.g., natal streams, downstream areas, and lentic habitats) at different times of year and for different durations prior to seaward migration, are expressed within species and populations of anadromous salmonids (Bourret et al., 2016). Juvenile life‐history expression, which ultimately determines juvenile habitat use, has been shown to be affected by both inter‐ and intraspecific interactions (Marco‐Rius et al., 2013) and environmental conditions (Bailey et al., 2018; Rich et al., 2009). This suggests that habitat requirements of salmonid populations can shift across productivity and environmental regimes, and that understanding how the expression of alternative life histories is affected by population density and environmental conditions can inform habitat requirements and consequently habitat management priorities.

We hypothesized that the production of juvenile anadromous salmonids exhibiting different LHPs, corresponding to different ages of migration from natal areas, varies in response to the density of conspecifics (Rich et al., 2009; Zimmerman et al., 2015). Juvenile salmon productivity most commonly exhibits negative density dependence (Einum et al., 2006; Grossman & Simon, 2020; Walters et al., 2013), but we predicted that a focus on individual LHPs would reveal greater complexity. Specifically, we predicted that we would detect positive density dependence in the prevalence of younger‐emigrating LHPs because more individuals should emigrate earlier in the presence of a larger number of conspecifics (Apgar et al., 2021). We predicted that we would find evidence for negative density dependence in the prevalence of older‐emigrating LHPs because density‐dependent emigration of younger individuals from the natal stream and density‐dependent mortality within the natal stream should reduce the number of individuals available to emigrate at older ages (Walters et al., 2013).

We also hypothesized that the production of different LHPs varies in response to critical environmental drivers (Sturrock et al., 2015, 2020). Streamflow has been identified as a positive and negative driver of juvenile salmon productivity (Apgar et al., 2021; Jones et al., 2020; Ohlberger et al., 2018; Warkentin et al., 2022), and we hypothesized that flow conditions would affect the production of alternative LHPs. If particular flow conditions are advantageous for growth and survival within natal habitat, fish may respond by choosing to remain for longer, and the number of surviving residents may increase (Scheuerell et al., 2020). Furthermore, flow may be a proximate trigger for migration, or may be directly related to a proximate trigger such as growth rate (Rich et al., 2009; Sturrock et al., 2015).

We evaluated evidence for our hypotheses by examining relationships between population density (for which we used female spawner density as an index), flow patterns, and the abundance of juvenile emigrants across four LHPs that we identified in a semelparous anadromous fish, Chinook salmon (*Oncorhynchus tshawytscha*), in a river basin in Washington State, USA. We found support for our hypotheses that density and flow affect the production of alternative LHPs. Our findings have implications for density‐dependent habitat use as well as population responses to environmental variability and change.

## 2 METHODS

### 2.1 Study system

**FIGURE 1:**
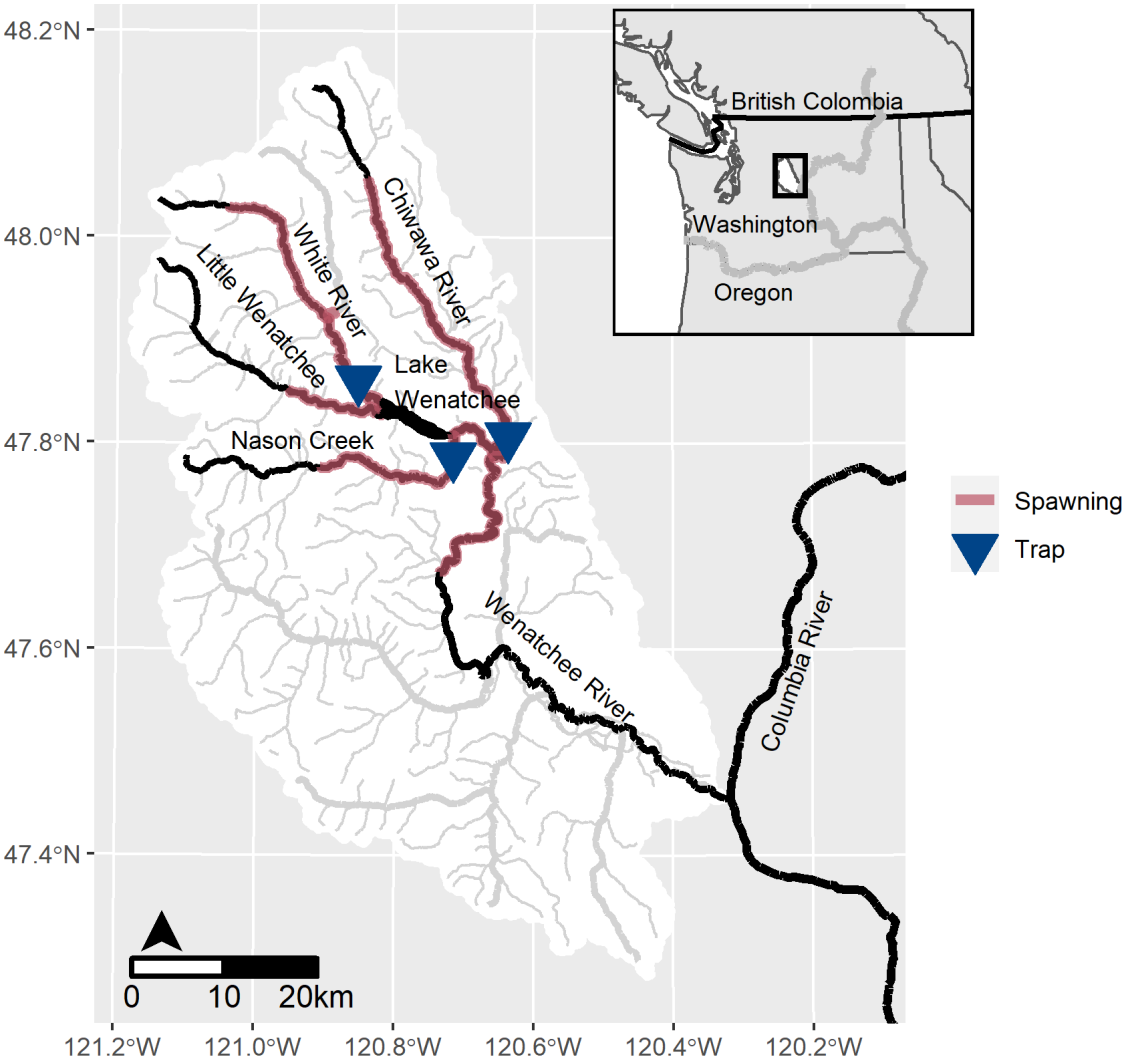
Wenatchee River basin, showing Chinook salmon spawning habitat in red and locations of rotary screw traps where out‐migrating juveniles were sampled as blue triangles.

The Wenatchee River is a tributary of the Columbia River (river kilometer, rkm, 754) located in Washington State (Figure 1), with a drainage area of roughly 3400 km^2^. The Wenatchee River basin supports a population of endangered Chinook salmon that is one of three populations within the Upper Columbia River Evolutionarily Significant Unit (ESU), one of 49 conservation units considered under the US Endangered Species Act (ESA). Spawning occurs in August–September and juveniles emerge from nests (redds) in winter. Juveniles exhibit a stream‐type life history, rearing within the Wenatchee River basin before emigrating to the marine environment in spring just over 1 year after eggs hatch. While there is little variability in the age of seaward migration, there is individual heterogeneity in age of emigration from natal streams, with some juveniles emigrating at younger ages to rear downstream prior to seaward emigration and others remaining in the natal stream until seaward emigration at age one (Buchanan et al., 2015).

We focused our analysis on monitoring data from three tributaries—the Chiwawa River, Nason Creek, and the White River (Figure 1)—that together comprise approximately 90% of the total spawning in the basin on average across years (Hillman et al., 2020). The hydrographs in these spawning streams are characterized by a substantial snowmelt‐driven spring freshet from April through July followed by summer low flows in August–September and a rainy season from October through December (Appendix S1: Figure S1). Peak water temperatures occur from July through September.

There are two conservation hatchery programs in the basin, which use returning natural‐ and hatchery‐origin adults as broodstock and release 1‐year‐old juveniles in the spring for their seaward migration. Because these hatchery juveniles migrate to the ocean very soon after release, they have little overlap with naturally produced juveniles within the Wenatchee River Basin. Furthermore, hatchery juveniles are marked such that they can be differentiated from naturally produced juveniles. However, the demographic connection between the natural and hatchery populations through the spawning of hatchery‐origin adults in the streams and the use of natural‐origin adults in the hatchery broodstock may influence the prevalence of alternative life histories in the natural population.

### 2.2 Data

#### 2.2.1 Juvenile data

Juvenile outmigrants were monitored with rotary screw traps operated near the mouth of the Chiwawa River (rkm 0.6) in 1997–2018, Nason Creek (rkm 1) in 2004–2018, and the White River (rkm 9) in 2006–2018 (Hillman et al., 2020). Traps were installed in early spring once ice in the river had melted, typically in early March, and were operated as continuously as possible until the river began to freeze again, typically in late November. Outages occurred periodically during the trapping season due to technical difficulties such as debris jamming the trap, very high discharges, or large releases of hatchery juveniles.

There were two components of the data collected at the rotary screw traps by the Washington Department of Fish and Wildlife and Yakama Nation Fisheries. The first component was the daily numbers of natural‐origin fish captured. During the spring, the traps captured both yearlings en route to the ocean and recently emerged subyearlings (i.e., alevin or fry), while in summer and fall, only subyearlings (i.e., parr) were captured. Subyearlings and yearlings were differentiated in spring based on length and date of capture (Appendix S2). The second component of the trap data was from mark‐recapture experiments that were conducted on several days each year to assess capture probabilities in each trap. In these experiments, captured fish >60 mm were marked with passive integrated transponder (PIT) tags and released 0.8–2.6 km upstream of the traps (Hillman et al., 2020). Fish <60 mm, which included most of the subyearling emigrants in spring and a portion of subyearling emigrants in summer, were not large enough to be safely tagged. The numbers of fish released upstream of the traps and the numbers of fish that were recaptured at the traps were recorded.

#### 2.2.2 Spawner data

The annual abundances of female spawners were estimated by walking spawning streams and counting redds (Hillman et al., 2020). Streams were surveyed every 7–10 days from late July through September, and redds were geo‐referenced to avoid double‐counting. All known Chinook salmon spawning habitat was surveyed by Washington Department of Fish and Wildlife and Chelan County Public Utility District. The counts of female spawners include both natural‐origin and hatchery‐origin spawners. Female spawner abundance data, which provide an index of the juveniles that these females produce, and thus the density of conspecifics experienced by these juveniles, were used to fit the stock‐recruitment model described below to evaluate density dependence in juvenile recruitment.

#### 2.2.3 Discharge data

We downloaded daily average stream discharge recorded at the US Geological Survey's Chiwawa River gauge using the *dataRetrieval* package in R (Hirsch et al., 2015). Daily average discharge in Nason Creek and the White River were recorded at Washington Department of Ecology stream gauges (Washington Department of Ecology, 2020).

### 2.3 Modeling framework

Evaluating environmental effects on juvenile abundance and LHP prevalence required three steps: (1) estimation of the daily abundance of juvenile emigrants past rotary screw traps, (2) summation of daily‐emigrant abundances within discrete LHPs, and (3) fitting models of the annual abundance of each LHP in each stream as a function of spawner abundance and environmental factors.

### 2.4 Step 1: Model of daily juvenile emigrants

#### 2.4.1 Process component

We modeled the daily abundance of yearling and subyearling emigrants passing screw traps at the mouths of natal streams separately, due to the break in the catch data during winter. We also modeled emigrant abundances from each natal stream separately because fish behavior and trap efficiency differed among them. We modeled the partially observed true number of daily emigrants $m_{t,y,s,a}$ on day t, year y, stream *s*, and age *a* on the log scale (so that abundance remained positive) as a function of a year‐specific average, $\mu_{y,s,a}^{m}$, the average day‐to‐day variation in log emigrant abundance across years, $\delta_{t,s,a}^{m}$, and year‐specific deviations of the daily‐emigrant abundance around the cross‐year daily average, $\epsilon_{t,y,s,a}^{m}$,

(1) $$\log\left(\;m_{t,y,s,a}\right)=\mu_{y,s,a}^{m}+\delta_{t,s,a}^{m}+\epsilon_{t,y,s,a}^{m}$$

Because the across‐year average daily errors, $\delta_{t,s,a}^{m}$, represented seasonality in emigration, which we assumed would be a non‐stationary process, we modeled them as a random walk, $\delta_{0,s,a}^{m}=0,{\;\delta }_{t,s,a}^{m}∼N\left(\delta_{t-1,s,a}^{m},\sigma_{s,a}^{\delta }\;\right)$. In contrast, the year‐specific daily errors, $\epsilon_{t,y,s,a}$, represent variation around the average, which we assumed would be stationary. We therefore modeled them as a stationary first‐order autoregressive process,

(2) $$\epsilon_{0,y,s,a}^{m}∼N\left[0,\sqrt{\frac{\sigma_{s,a}^{\epsilon }}{1-{\rho_{s,a}^{\epsilon }}^{2}}}\right]$$

(3) $$\epsilon_{t,y,s,a}^{m}=\rho^{\epsilon }\epsilon_{t-1,y,s,a}^{m}+\lambda_{t,y,s,a},\;\lambda_{t,y,s,a}∼N\left(0,\sigma_{s,a}^{\epsilon }\right)$$

with autocorrelation coefficient $\rho_{s,a}^{\epsilon }$ and innovation standard deviation $\sigma_{s,a}^{\epsilon }$.

#### 2.4.2 Observation component

Observed catches $c_{t,y,s,a}$ were assumed to follow a negative binomial distribution, which allows for greater observation error than a Poisson distribution. The expected value of daily catch, $\mu_{t,y,s,a}^{c}=m_{t,y,s,a}\;p_{t,y,s,a}$ was equal to the product of the latent daily‐emigrant abundance, $m_{t,y,s,a}$, and the daily capture probability $p_{t,y,s,a}$, The variance was equal to $\mu_{t,y,s,a}^{c}+\frac{{\mu_{t,y,s,a}^{c}}^{2}}{\phi_{s,a}}$, where $\phi_{s,a}$ was a scale parameter which was restricted to be positive and was assumed to be constant across years.

The capture probability of fish emigrating past a trap was modeled on the logit scale as a function of daily discharge:

(4) $$\text{logit}\left(p_{t,y,s,a}\right)=\iota_{y,s,a}+\kappa_{y,s,a}D_{t,y,s,a}+\nu_{w\left(t\right),s,a}+\xi_{w\left(t\right),y,s,a},$$

where $\iota_{y,s,a}∼N\left(\beta_{0,s,a}^{p},\;\sigma_{s,a}^{\iota }\right)$ is a random intercept for each year, $\kappa_{y}\sim N\left(\beta_{1,s,a}^{p},\;\sigma_{s,a}^{\kappa }\right)$ is a random effect of *Z*‐scored daily stream discharge ($D_{t,y,s,a}$) in each year, $\nu_{w\left(t\right),s,a}∼N\left(0,{\;\sigma }_{s,a}^{\nu }\right)$ is a random effect of week (*w*) that is common across years, and $\xi_{w\left(t\right),y,s,a}∼N\left(0,\sigma_{s,a}^{\xi }\right)$ is a random effect of week specific to each year.

The capture probability, $p_{t,y,s,a}$, was informed by the mark‐recapture dataset. The number of fish recaptured, $k_{t,y,s,a}$, out of the number of marked fish released upstream of the trap, $n_{t,y,s,a}$, was assumed to be binomially distributed, $k_{t,y,s,a}∼\mathrm{Bin}\left(n_{t,y,s,a},p_{t,y,s,a}\right)$.

We modeled the capture probability at the screw trap of all marked fish released upstream of the trap based on stream discharge on the day of release. While fish were recaptured up to 4 days after release, and these fish could have experienced some variability in daily stream discharge between release and recapture, 91.5% of recaptured fish were recaptured on the date that they were released. Therefore, while our assumption may induce some bias in our estimates, the bias is expected to be small, and this simplifying assumption was made to reduce model complexity. We assumed that all fish of a given age (subyearling and yearlings) and stream had the same capture probabilities on a given day, including those <60 mm, which were too small to be safely tagged.

### 2.5 Step 2: Juvenile life‐history delineation

The average time series of the (partially observed) daily‐emigrant abundances across streams and brood years contained four modes, indicating that emigrants could be categorized into four alternative LHPs of emigration timing from natal streams (Appendix S1: Figure S2). To delineate the LHPs, we fit a four‐component normal mixture distribution to the time series of emigrant abundances, rounded to the nearest integer, from each brood across all years and natal streams using the package *mixtools* in the R statistical environment (Benaglia et al., 2009; R Core Team, 2021). We then determined the days of the year corresponding to local minima between the modes of the mixture distribution components, which were used as cutoffs to delineate the four windows of emigration corresponding to alternative LHPs. Thus, it was assumed that the emigration time windows that defined the LHPs were the same across streams and years. Daily‐emigrant abundances were summed within periods to calculate the total number of emigrants ($M_{h,y,s}$) from brood year *y* and stream *s* expressing LHP *h*,

(5) $$M_{h,y,s}=\sum_{t=t_{0,h}}^{t_{f,h}}m_{t,y,s,a},$$

where $t_{0,h}$ was the first day of the time window corresponding with LHP *h* and $t_{f,h}$ was the final day. We assumed there was no emigration during the winter period when traps were never operated. While this leads to underestimation of total emigrant abundances, it should not bias our inference about correlates of interannual variation in life‐history prevalence. We expected the number of emigrants in winter to be relatively small, considering the relatively small number of emigrants observed in late fall prior to trap removal and in early spring just after trap installation.

### 2.6 Step 3: Spawner to juvenile emigrant model

#### 2.6.1 Process component

To model the relationship between the abundance of female spawners, which provide an index of juvenile density, and the juvenile emigrants that they produced, we fit the Myers et al. (1995) version of the classic Beverton–Holt model, which can simultaneously describe positive and negative density dependence,

(6) $$J_{h,y,s}=\frac{\alpha_{h,s}S_{y\left(h\right),s}^{\gamma_{h,s}}}{1+\frac{\alpha_{h,s}S_{y\left(h\right),s}^{\gamma_{h,s}}}{J_{h,s}^{\max}}}\exp\left(\epsilon_{h,y,s}^{J}\right),$$

where $J_{h,y,s}$ is the abundance of juvenile emigrants expressing LHP *h* produced by female spawners $S_{y\left(h\right),s}$ in year $y\left(h\right)$—which is 1 year previous to the emigration year for subyearling emigrants and 2 years previous for yearling emigrants—and stream *s*. Note that $J_{h,y,s}$ is in theory the same quantity as $M_{h,y,s}$ from the model of daily juvenile abundances. However, we fit the models separately, developing log‐normal distributions of $M_{h,y,s}$ with a parametric bootstrap of the model of daily juvenile abundances and using it as a penalty in the spawner‐recruit model (see “Observation component” below). Spawner and juvenile abundance were scaled by the length (km) of spawning habitat in each stream to facilitate hierarchical modeling across streams. In the modified Beverton–Holt model, $\alpha_{h,s}$ cannot be interpreted as the maximum productivity, as it can in the classic Beverton–Holt, whereas $J_{h,s}^{\max}$ retains its interpretation as the maximum expected juvenile emigrant abundance. The generalized model includes positive density dependence when $\gamma_{h,s}$ >1.0 and negative density dependence when $\gamma_{h,s}$ <1.0. We allowed for multiplicative log‐normal residual error $\epsilon_{h,y,s}^{J}$, which we modeled using a latent variable model described below.

The three shape parameters of the modified Beverton–Holt model (Equation 6) were not well informed by the data for all LHPs and streams, so we modeled them hierarchically across streams for each LHP. We modeled $\alpha_{h,s}$, $\gamma_{h,s}$, and $J_{h,s}^{\max}$ hierarchically such that $\alpha_{h,s}∼\text{lognormal}\;\left(\mu_{h}^{\alpha },\sigma_{h}^{\alpha }\right)$, with equivalent expressions for $\gamma_{h,s}$ and $J_{h,s}^{\max}$. Because there were only three streams available to estimate the hyperdistribution standard deviations $\sigma_{h}^{\alpha }$, we applied exponential regularizing penalties [e.g., $\sigma_{h}^{\alpha }∼\exp\;\left(\lambda \right)$] (Simpson et al., 2017), a common practice when fitting random‐effect distributions with few levels (Gelman, 2006). We applied the same amount of penalization (i.e., common λ) for the three different parameters and four LHPs.

To make inference about density dependence, we penalized deviations of the log means of the hyperdistributions of $\gamma_{h,s}$ and $J_{h,s}^{\max}$ from values that would result in density‐independent production. The modified Beverton–Holt model simplifies to a density‐independent model when γ=1 and $J^{\max}\to \infty$. Therefore, we applied the following penalties on the log means of the hyperdistributions of these parameters:

(7) $$\mu_{h}^{\gamma }∼N\left(0-\frac{{\tau_{h}^{\gamma }}^{2}}{2},\tau_{h}^{\gamma }\right),\tau_{h}^{\gamma }∼\exp\left(\lambda^{\gamma }\right),$$

(8) $$\mu_{h}^{J^{\max}}∼N\left(\log\left(\nu^{J^{\max}}\right)-\frac{{\tau_{h}^{J^{\max}}}^{2}}{2},\tau_{h}^{J^{\max}}\right),\tau_{h}^{J^{\max}}∼e\mathrm{xp}\left(\lambda^{J^{\max}}\right)$$

which puts weight on a model with minimal density dependence when $\nu^{J^{\max}}$ is large. This approach is an alternative to multimodel inference via model selection (e.g., based on AIC; Burnham & Anderson, 2004), because it favors parsimony and relies on the data to provide support for additional model complexity (i.e., density dependence). We set $\nu^{J^{\max}}$ at 1.5e4, which was greater than the upper limit of the 95% confidence interval of the largest observed abundance of emigrants in the dataset (1.26e4). We applied the same penalty rates (i.e., $\lambda^{\gamma }$and $\lambda^{J^{\max}}$) for all LHPs.

We allowed for correlated process errors, $\epsilon_{h,y,s}^{J},$ to account for the structural relationship whereby the number of emigrants expressing each LHP will affect the number of juveniles available to emigrate in subsequent periods. Correlated errors also account for variability in survival within the natal stream affecting the abundance of multiple LHPs. To do so, residual process errors $\epsilon_{h,y,s}^{J}$, were modeled using a latent variable model (Warton et al., 2015),

(9) $$\epsilon_{h,y,s}^{J}=x_{h,y,s}^{\prime }\beta_{h}^{\epsilon }+\omega_{y}l_{h,s}+\eta_{h,y,s},$$

where $x_{h,y,s}$ are environmental covariates, $\beta_{h}^{\epsilon }$are regression coefficients, $\omega_{y}∼N\left(0,1\right)$ is a latent variable, $l_{h,s}$ are life‐history‐ and stream‐specific coefficients for the latent variable (i.e., a factor loading), and $\eta_{h,y,s}∼N\left(0,\sigma_{h,s}^{\eta }\right)$ is an error that is independent among LHPs and natal streams. We placed a regularizing penalty on independent error standard deviations, $\sigma_{h,s}^{\eta }∼\exp\left(\lambda^{\eta }\right)$, to help with convergence by placing relatively less weight on very large or small independent error variances.

We used spawners as an index of the density that age‐0 juveniles would experience due to other members of their cohort; however, for a period in spring, age‐0 juveniles also share habitat with age‐1 juveniles, which could also exert an effect via density‐dependent mechanisms. To evaluate whether the density of age‐1 juveniles from the previous cohort, which overlap with the age‐0 juveniles during spring, affected juvenile production, we explored whether the abundance of female spawners that produced the previous cohort was correlated with process errors. Finding no evidence of such a relationship in exploratory analyses, we concluded that there were minimal density‐dependent interactions between juveniles of subsequent cohorts and did not include indices of previous cohorts' density as covariates in the model.

To assess how interannual variability in flow patterns affected survival and movement, we evaluated the effect of stream discharge on process errors (Equation 9). We included the log maximum of daily average stream discharge during a brood year's first winter (November–February), when eggs and juveniles are susceptible to mortality and displacement by scouring high flows (Clark et al., 2001), as a covariate on process errors in the production of each LHP. The mean of daily discharge during summer (June–September), which affects total habitat availability (Scheuerell et al., 2020), was included as a covariate on each LHP except spring subyearling emigrants, which emigrate before summer. Finally, we included log maximum winter discharge during the second winter of a year class, which can dislocate habitat and juveniles (Scheuerell et al., 2020), as a covariate on the spring yearling LHP only. Relationships between process errors and stream flow covariates ($\beta_{h}^{\epsilon }$) were assumed to be the same for a given LHP across streams. That is, we assumed that deviations from average discharge within a given stream affected fish expressing a specific LHP in the same way across streams. We therefore *Z*‐scored the discharge covariates for each stream using the stream‐specific mean and standard deviation, such that we were modeling the effects of standardized annual deviations from within‐stream average discharge.

#### 2.6.2 Observation component

For computational efficiency, the models of daily juvenile abundance (informed by screw‐trap data) and the spawner‐recruit models (informed by the summed estimates of juvenile abundance) were fitted in two stages. Data on the abundance of juvenile emigrants and spawners entered the likelihood as log‐normal penalties. The estimates of log‐mean emigrant abundance for a given LHP, stream, and brood year, $\bar{M_{h,y,s}^{*}}$, were developed from the model of daily juvenile emigrants via a parametric bootstrap described below, and were assumed to be normally distributed around the log of the latent emigrant abundance in the model of LHP abundance (Equation 6),

(10) $$\bar{M_{h,y,s}^{*}}\sim N\left(\log\left(J_{h,y,s}\right),\sigma_{h,y,s}^{\bar{M^{*}}}\right),$$

with observation error $\sigma_{h,y,s}^{\bar{M^{*}}}\;$equal to the standard deviation from the parametric bootstrap of the daily juvenile abundance model.

To conduct the parametric bootstrap, we drew 10,000 parameter sets from a multivariate normal distribution defined by maximum likelihood estimates of parameters and their covariance matrix obtained by inversion of the Hessian matrix calculated using Template Model Builder (TMB) (Kristensen et al., 2016) in R. For each bootstrap sample, we calculated values of $M_{h,y,s}$using Equations 1 and 5, and calculated their log‐mean, $\bar{M_{h,y,s}^{*}}$, and log‐standard deviation, $\sigma_{h,y,s}^{\bar{M^{*}}}$.

The observed redd counts were assumed to be log‐normally distributed around the true female spawner abundances, with standard deviation equal to 0.1:

(11) $$S_{y,s}^{\mathrm{obs}}∼\text{logNormal}\left(\log\left(S_{y,s}\right),0.1\right).$$

The observation standard deviation of 0.1 was chosen based on information from Murdoch et al. (2019), and equates to a coefficient of variation of approximately 10%.

### 2.7 Parameter estimation

Parameters in all models were estimated using a mixed‐effects framework. Specifically, parameters were estimated by expectation maximization where marginal likelihoods were calculated by TMB using Laplace approximation to integrate over random effects. Optimization was carried out using the *TMBhelper* package in R (Thorson, 2020). The daily errors $\delta_{t}^{m}$ and $\epsilon_{t,y}^{m}$ in the daily‐emigrant model, and $\iota_{y},\kappa_{y},\nu_{w\left(t\right)}\text{and}\;\xi_{w\left(t,y\right)}$ in the capture‐probability model were treated as random effects. The fixed effects were $\mu_{y}^{m},\rho^{\epsilon },\sigma^{\epsilon }$ and $\sigma^{\delta }$ in the daily‐emigrant model and $\beta^{p}$, $\sigma^{\nu },\sigma^{\xi },\text{and}\;\phi$ in the observation model. In the spawner‐recruit model, the log‐transformed latent spawner abundances $\log\left(S_{y,s}\right)$, latent variables $\omega_{y}$, idiosyncratic errors $\eta_{h,y,s}$, log means of the shape‐parameter hyperdistributions $\mu_{h}^{\gamma }$ and $\mu_{h}^{J^{\max}}$, and stream‐specific shape parameters $\alpha_{h,s}$, $\gamma_{h,s}$, and ${J^{\max}}_{h,s}$ were treated as random effects. The fixed effects were $\mu_{h}^{\alpha }$, $\sigma_{h}^{\alpha },\sigma_{h}^{\gamma },\sigma_{h}^{J^{\max}}$, λ, $\tau_{h}^{\gamma },\tau_{h}^{J^{\max}}$, $\lambda^{\gamma }$, $\lambda^{J^{\max}},\beta_{h}^{\epsilon }$, $l_{h,a}$, $\sigma_{h,s}^{\eta }$, and $\lambda^{\eta }$.

## 3 RESULTS

Across streams and years, there were four largely distinct modes of juvenile emigration from each brood, although certain modes were less pronounced in individual streams (Appendix S1: Figure S2). These four modes included: (1) subyearling emigrants during the ascending limb of the spring freshet in March–April (hereafter *spring subyearlings*), (2) subyearling emigrants in May–September as flows were declining and water temperatures increasing (*summer subyearlings*), (3) subyearling emigrants from October–November coinciding with decreasing water temperature and rain‐driven increases in discharge (*fall subyearlings*), and (4) yearlings emigrating in March–June, on the ascending limb of the spring freshet (*spring yearlings*) en route to the marine environment.

**FIGURE 2:**
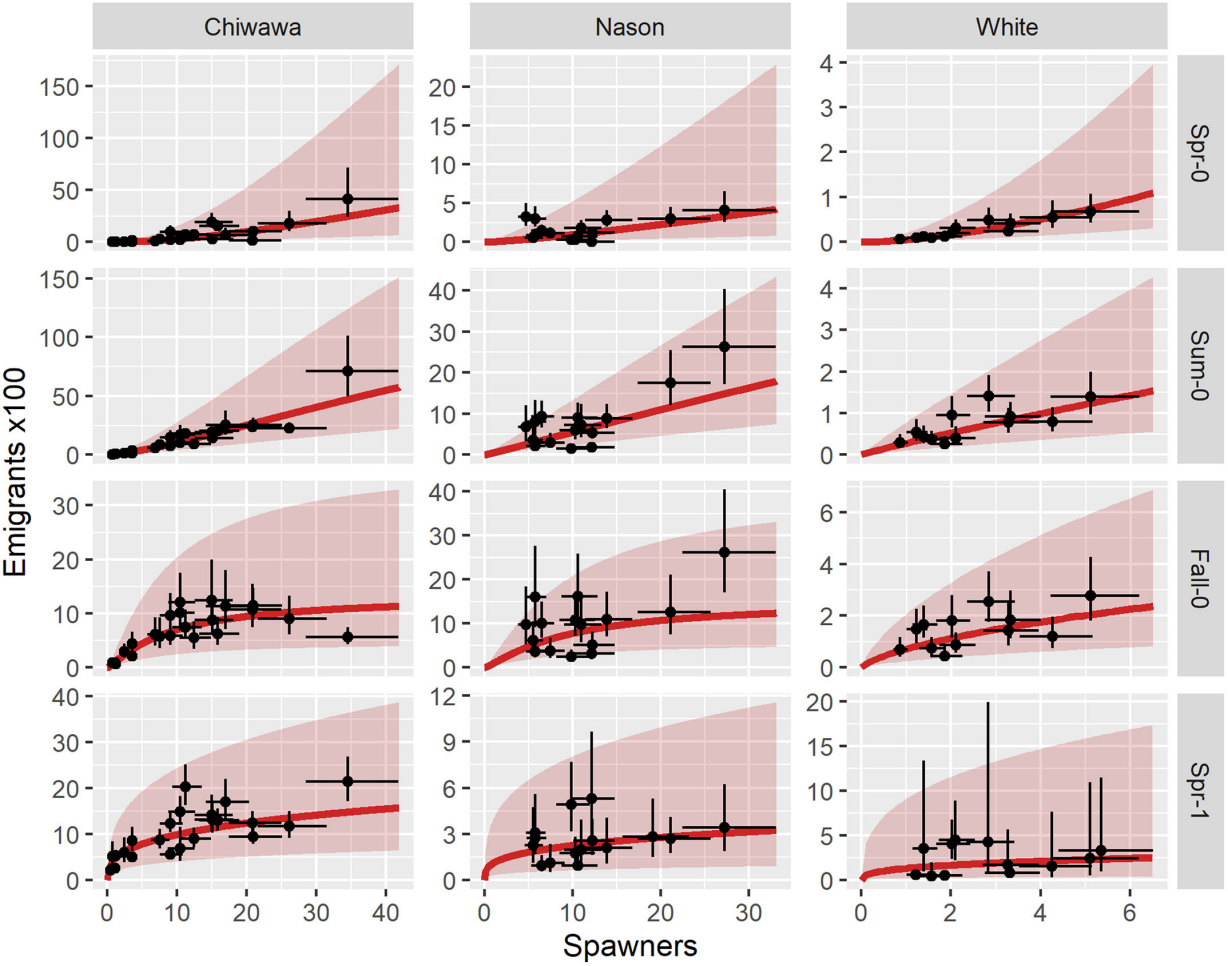
Functional relationships between spawner and juvenile emigrant abundances per kilometer of spawning habitat by natal stream and juvenile life history, where the life histories could be; *Spr‐0* = spring subyearling emigrants, *Sum‐0* = summer subyearlings, *Fall‐0* = fall subyearlings, and *Spr‐1* = spring yearling emigrants. The functional form is a modified Beverton–Holt model. Points represent estimates of spawner and juvenile emigrant abundances with 95% confidence intervals. The red lines represent the expected number of juvenile emigrants for a given spawner abundance. The red envelope is the 95% prediction interval representing process error.

**FIGURE 3:**
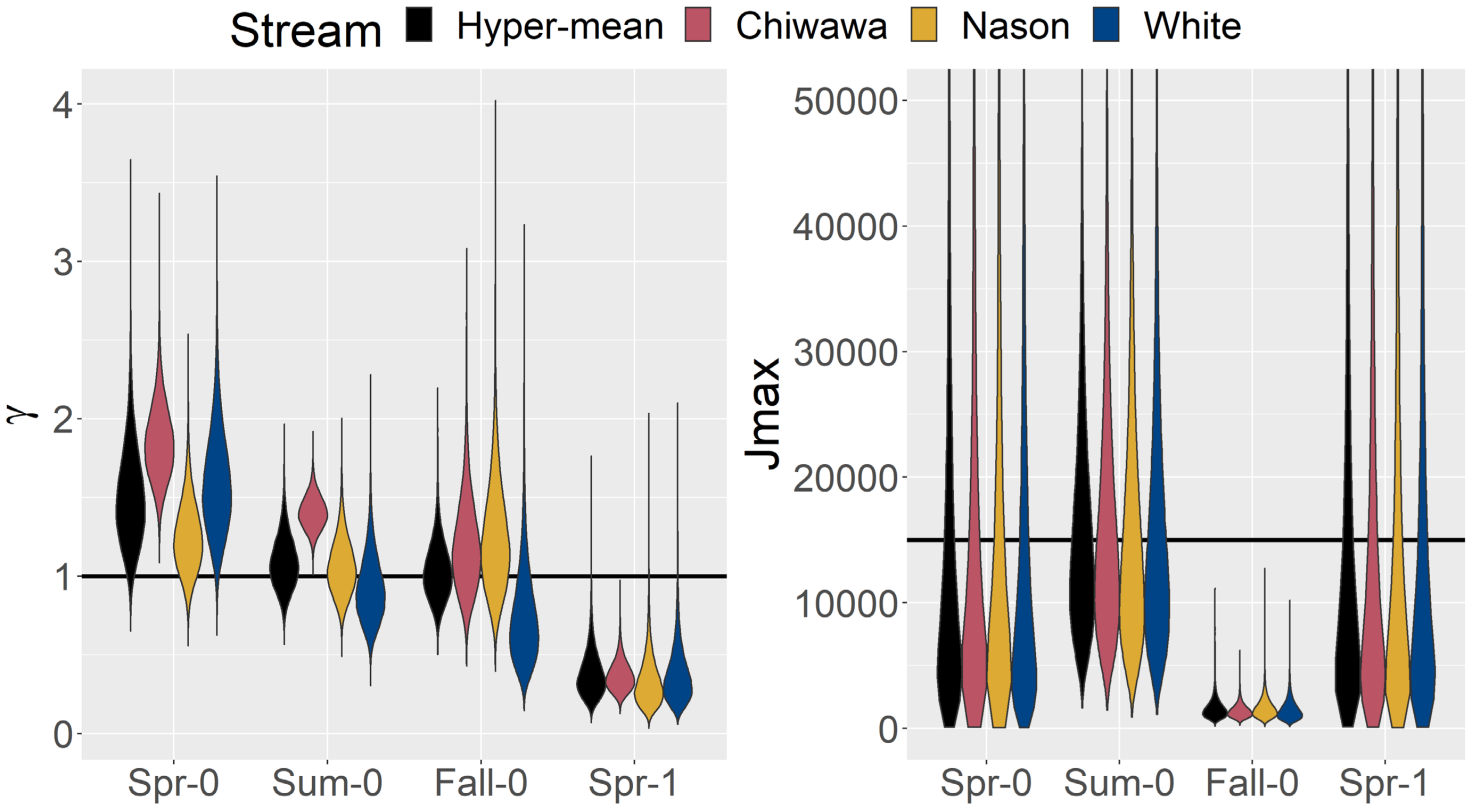
Estimates of density‐dependent parameters *γ* and *J* ^max^ in the functional relationship between spawner and juvenile emigrant abundance by juvenile life‐history pathway and natal stream. The solid horizontal lines represent the expected values of the parameters in the absence of density dependence and given the penalties assessed in the model. For *γ*, values <1 induce negative density dependence and values >1 induce positive density dependence. Thus, the fitted *γ* values suggest positive density dependence in the Spr‐0 life‐history pathway and negative density dependence in Spr‐1. For *J* ^max^, which represents the maximum expected abundance of juveniles per kilometer of natal stream habitat, the expected value of 15,000 juveniles was twice the estimate of the maximum number of juveniles observed in any year of monitoring. The diffuse distributions of *J* ^max^ for Spr‐0, Sum‐0, and Spr‐1 provide little evidence of negative density dependence, whereas the tighter distributions around smaller values for Fall‐0 provide evidence of negative density dependence.

The estimates of γ and *J* ^max^ parameters provided evidence of positive density dependence in the production of spring subyearling production and to a lesser degree in summer subyearling production (Figures 2 and 3, Appendix S1: Table S1). The mean γ parameter, which induces positive density dependence when >1 and negative density dependence when <1, was 1.51 (95% CI = 1.03, 2.23) for spring subyearlings. All three streams had mean *γ* values that were greater than one, but confidence intervals overlapped one in two of three streams (Figure 3, Appendix S1: Table S1). For summer subyearling emigrants, the mean *γ* parameter was 1.09 (0.80, 1.47), providing little evidence of density dependence, although the *γ* parameters for summer subyearlings suggested positive density dependence in the Chiwawa River (Figure 3, Appendix S1: Table S1). In all streams, the *J* ^max^ parameters for spring and summer subyearlings were similar to the expectation based on the penalty, providing no evidence of negative density dependence (Figure 3, Appendix S1: Table S1).

**FIGURE 4:**
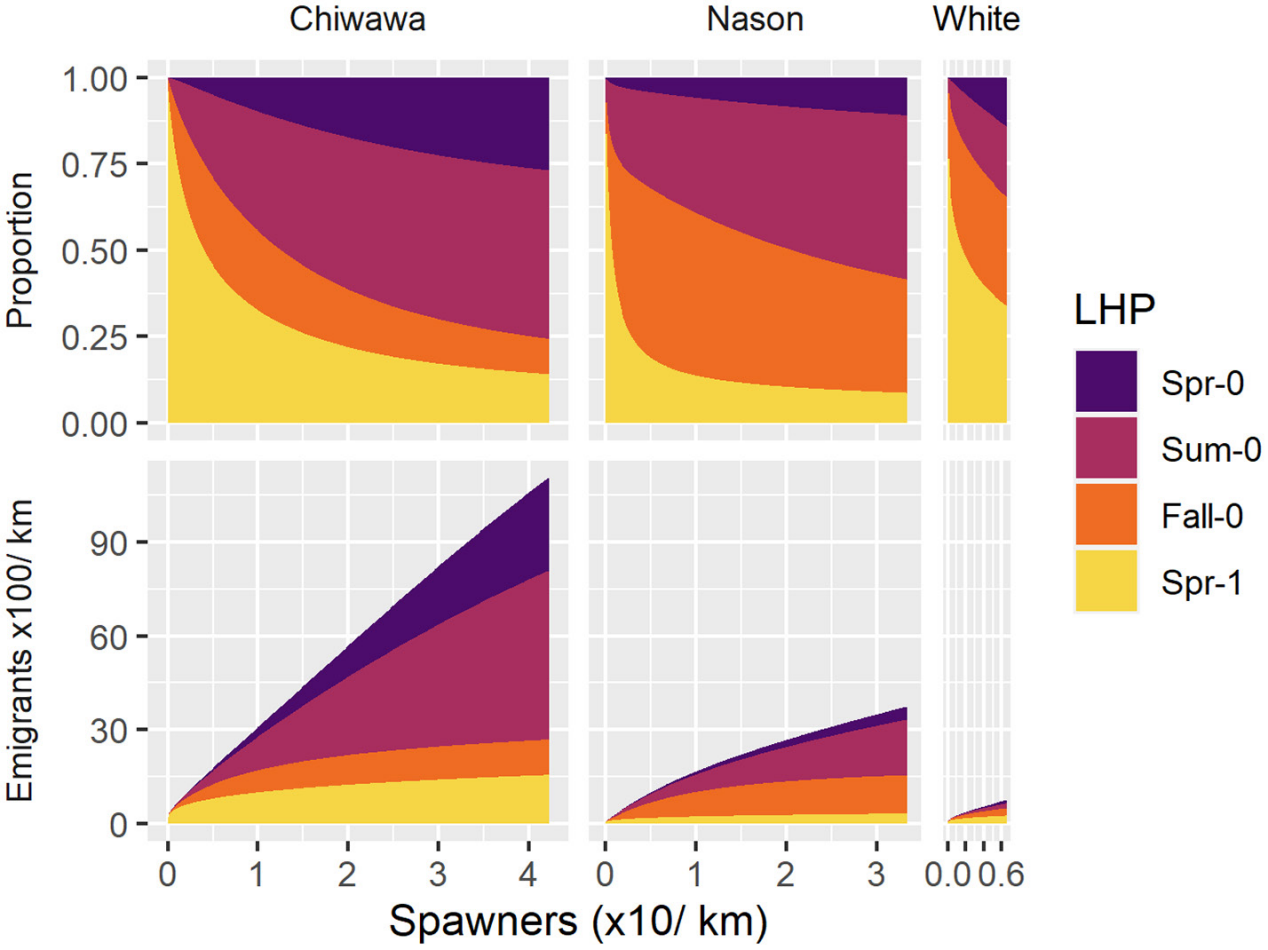
Proportion (top row) and expected abundance (bottom row) of juvenile emigrants expressing each of four juvenile life‐history pathways (LHPs) as a function of spawner abundance, based on models that account for density dependence in the production of each LHP. Predictions span the range of spawner abundance for which estimates of juvenile abundance were available to parameterize models. Estimates represent the expectations for an average year.

Negative density dependence was evident in fall subyearling and spring yearling emigrants. (Figures 2 and 3). The mean estimate of γ for fall subyearlings was 1.03 (0.73, 1.45), providing little evidence of density dependence. However, the mean estimate of *J* ^max^, the maximum expected juvenile abundance, for fall subyearlings was 1480 (597, 3706), much less than expected based on the penalty, suggesting negative density dependence. For spring yearling emigrants, negative density dependence was evident based on the average γ parameter of 0.37 (0.17, 0.79), considerably less than one. As a result of the positive density dependence in younger‐emigrating LHPs, and negative density dependence in older‐emigrating LHPs, younger emigrants comprised a greater proportion of all emigrants as spawner density increased (Figure 4).

**FIGURE 5:**
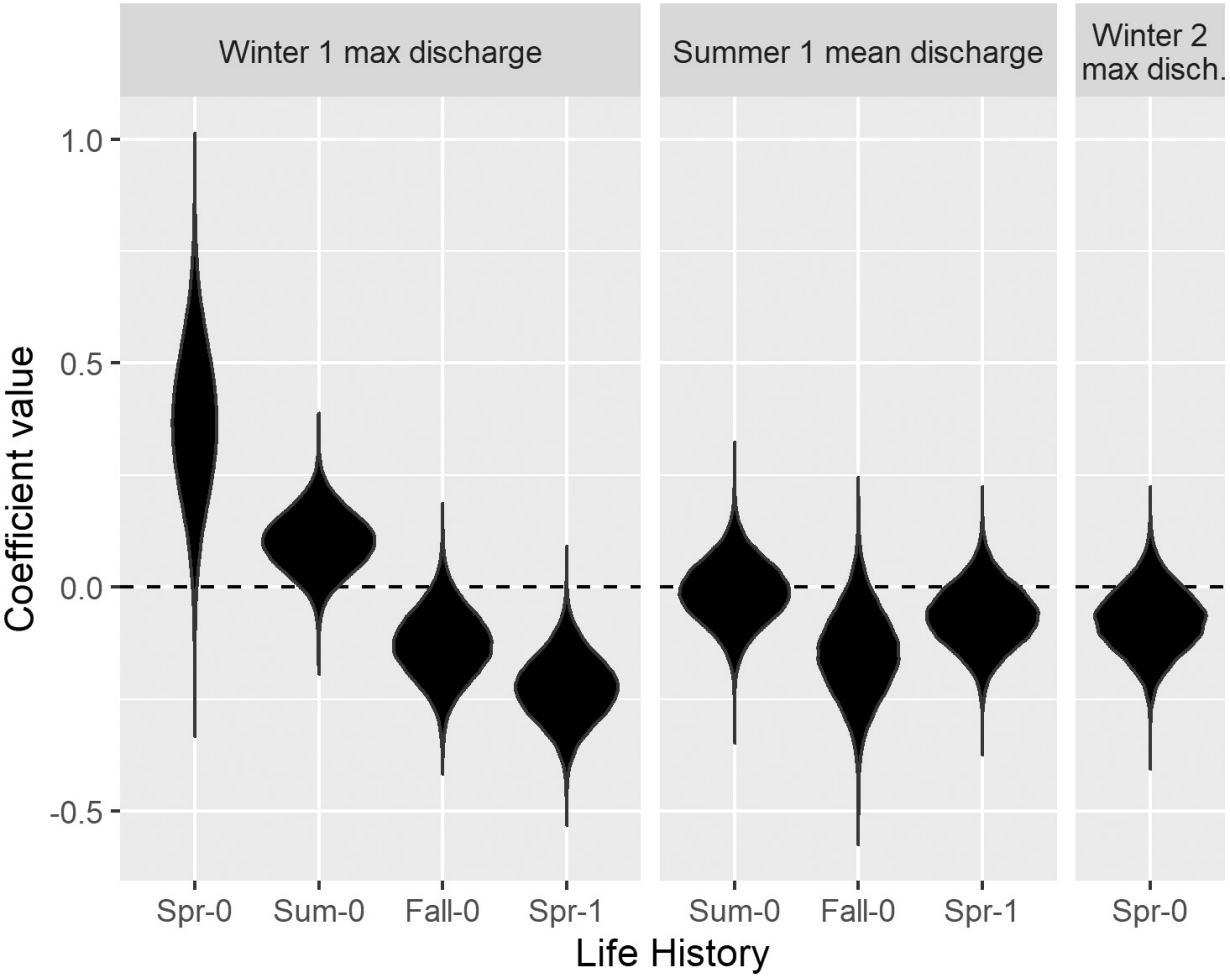
Coefficient values for the effects of log maximum daily stream discharge during a brood year's first winter (Winter 1), average discharge during its first summer (Summer 1), and log maximum discharge during its second winter (Winter 2) on process error in the annual abundance of each juvenile life‐history pathway.

Effects of stream discharge on process errors in abundance were variable among LHPs (Figure 5). Maximum daily stream discharge during each brood year's first winter was positively associated with process errors in the abundance of the younger spring (0.37; 0.04, 0.69) and summer (0.10; −0.03, 0.23) subyearling emigrants, and negatively associated with the abundance of the older fall subyearling (−0.13; −0.28, 0.02) and spring yearling (−0.22; −0.37, −0.08) emigrants. Average stream discharge during the summer was negatively associated with process error in fall subyearling emigrant abundance (−0.14; −0.33, 0.04).

## 4 DISCUSSION

We tested the hypothesis that population density influences the prevalence of alternative LHPs in an anadromous salmonid and found support for this hypothesis. We found evidence of positive density dependence in the production of younger emigrants and negative density dependence in the production of older emigrants. This suggests that positive density‐dependent emigration and potentially negative density‐dependent survival occurs in natal streams, resulting in a greater prevalence of younger emigrants as population size increases. Our evidence for positive density dependence in younger emigrants and negative density dependence in older emigrants is consistent with previous studies of juvenile salmon (Apgar et al., 2021; Einum et al., 2006; Thorson et al., 2014; Walters et al., 2013), providing further evidence that habitat needs change with population density. As the proportions of younger emigrants increase at higher population densities, the fate of younger emigrants in downstream rearing habitats should have a greater effect on overall population productivity. Therefore, functioning downstream habitats may be required to maintain larger population densities even if they are used by a relatively small component of the population at low population densities (Cordoleani et al., 2021; Sturrock et al., 2015). Furthermore, the effectiveness of hatchery supplementation in increasing the production of naturally produced juveniles may be enhanced by the availability of functional downstream rearing habitat.

We also found support for the hypothesis that environmental factors affect the production of alternative LHPs. We found evidence that the abundance of younger emigrants was greater than expected in years with above‐average stream flow during their first winter, prior to when most individuals emerge from the gravel, whereas the abundance of older emigrants was negatively associated with first‐winter stream flow. This is consistent with Sturrock et al. (2015) who found that younger emigrants contributed more to adult returns in wetter years. Higher winter flows are associated with warmer temperatures (Mantua et al., 2010), which are in turn associated with earlier emergence, faster growth rates (Beer & Steel, 2018; Sparks et al., 2017), and younger emigration from natal rearing areas (Bradford & Taylor, 1997; Cline et al., 2019; Rich et al., 2009; Sloat & Reeves, 2014), leaving fewer fish remaining to emigrate at older ages. Winter temperature and discharge are projected to increase with climate change (Mantua et al., 2010), suggesting that younger‐emigrating LHPs may become more common in the future, based on the relationship between winter discharge and LHP prevalence that we observed. Therefore, there could be a growing number of juveniles using downstream rearing habitats in the future.

We also found that summer stream flow was negatively associated with the prevalence of fall subyearling emigrants. One explanation is that higher summer flows were associated with greater snowpack and lower stream temperatures (Mantua et al., 2010), which decrease growth rates and the propensity of individuals to disperse from natal habitats as opposed to remaining until the following spring (Bradford & Taylor, 1997; Rich et al., 2009; Sloat & Reeves, 2014). However, we did not detect a positive effect of summer stream flow on the abundance of age‐1 emigrants. Nason Creek generally had the warmest average summer temperatures (Isaak et al., 2016) of the three natal streams (Appendix S1: Figure S1) in this study and produced the highest proportions of fall subyearling emigrants (Figure 4), supporting our expectation that lower stream temperatures reduce growth rates and emigration propensity. Snowpack is projected to decrease and summer stream temperatures to increase in the future (Mantua et al., 2010), which could lead to increased prevalence of the fall subyearling LHP that overwinters in downstream habitats.

Our results add more support for the hypothesis that environmental conditions such as streamflow affect life‐history prevalence, contributing to variability in habitat use through time. This phenomenon may help explain previously described patterns of variability in the relative productivity of alternative juvenile‐rearing habitats within river basins through time (Brennan et al., 2019; Phillis et al., 2018). Consequently, maintenance of a portfolio of functional habitat areas could contribute to population stability through time. Further analyses could explore interactions between density and streamflow covariates by evaluating environmental covariate effects on shape parameters in the Beverton–Holt model.

Further understanding the genetic, ontogenetic, and behavioral factors that drive life‐history expression could inform how climate change and hatchery supplementation might affect juvenile salmon survival and dispersal. Experimental evidence suggests that the tendency of Chinook salmon to distribute downstream may be positively associated with egg size (Thorn & Morbey, 2018), body size (Bradford & Taylor, 1997; Cogliati et al., 2018), and growth rate (Sloat & Reeves, 2014). Egg size can be affected by hatchery supplementation (Heath et al., 2003), while growth rate and body size are affected by the interaction of genotype with conditions such as water temperature (Beer & Steel, 2018). Future consideration of eco‐evolutionary drivers of juvenile LHP expression could help predict population responses to climate‐change impacts on thermal and flow regimes, and the multi‐generational effects of introgression between hatchery and wild populations (Vindenes & Langangen, 2015).

With the data available, we were not able to separate survival and emigration, which simultaneously contribute to the abundance of each LHP. Separating these processes would require direct information on survival within natal streams across time. Therefore, our model is not mechanistic in modeling the multiple demographic processes that generate emigrant abundances. Rather, our model of the emergent patterns provides insights into the drivers of juvenile life‐history diversity that have value for understanding how habitat use changes with population density and environmental variability.

This study furthers our understanding of how population density affects life‐history prevalence within populations. Similar to our finding that early migration increases at higher densities, Martin et al. (2022) found that a partially migratory population of elk conformed to Ideal Free Distribution theory, where animals select habitats that maximize individual fitness. According to this theory, as densities in the natal stream increase, juvenile salmonids should increasingly select habitats with lower densities to access the resources they need for growth prior to migration. Furthermore, the effects of population density on life‐history prevalence can be mediated by environmental factors (Brown, White, & Peet, 2021; Diez et al., 2021), and the effects of environmental drivers on life‐history prevalence may increase near carrying capacity (Jesmer et al., 2021).

Identifying drivers of life‐history variability improves understanding of critical factors that, in combination with the fitness of alternative life‐history pathways, determine population dynamics (Raffard et al., 2019) and may help predict responses to environmental change (Berger et al., 2017; Sturrock et al., 2020). Intraspecific diversity in life‐history traits directly affects habitat use (Phillis et al., 2018) and has been shown to mediate the effects of competition and environmental variability on population vital rates like growth, survival, and reproduction (Duffy, 2010; Jenouvrier et al., 2015; Zaiats et al., 2021), contributing to population stability and resiliency (Schindler et al., 2010). Therefore, accounting for life‐history diversity in population models could improve predictions of demographic effects driven by climate change, management, and other factors (Cunningham et al., 2020; Marco‐Rius et al., 2013; Phillips et al., 2017).

## AUTHOR CONTRIBUTIONS

**Mark Sorel:** Conceptualization (equal); data curation (equal); formal analysis (lead); methodology (equal); software (equal); writing – original draft (lead); writing – review and editing (equal). **Andrew R Murdoch:** Conceptualization (equal); data curation (supporting); writing – review and editing (equal). **Richard W Zabel:** Conceptualization (equal); formal analysis (supporting); funding acquisition (equal); methodology (supporting); project administration (supporting); writing – review and editing (equal). **Cory Kamphaus:** Data curation (equal); resources (equal); writing – review and editing (supporting). **Eric R Buhle:** Formal analysis (supporting); methodology (supporting); software (supporting); writing – review and editing (equal). **Mark D Scheuerell:** Formal analysis (supporting); methodology (supporting); writing – review and editing (equal). **Sarah Converse:** Conceptualization (equal); formal analysis (equal); funding acquisition (lead); methodology (equal); project administration (lead); supervision (lead); writing – original draft (supporting); writing – review and editing (equal).

## CONFLICT OF INTEREST STATEMENT

The authors have no conflicts of interest to declare.

## Supporting information


Appendix S1.

Appendix S2.

## Data Availability

Data and code (Sorel et al., 2023) are available from Zenodo: https://doi.org/10.5281/zenodo.7876213.
