# Supplementary material for: Effects of population density and environmental conditions on life‐history prevalence in a migratory fish
Source: Ecol Evol. 2023 May 23;13(5):e10087. doi: 10.1002/ece3.10087 (PMC10206029; doi:10.1002/ece3.10087)
Supplement: Supplementary file 1 — Appendix S1. [file ECE3-13-e10087-s001.docx]

# Appendix S1

Effects of population density and environmental conditions on life history prevalence in a migratory fish

Mark H. Sorel^1^, Andrew R. Murdoch^2^, Richard W. Zabel^3^, Cory M. Kamphaus^4^, Eric R. Buhle^3,5^, Mark D. Scheuerell^6^, and Sarah J. Converse^7^

Table S1. Parameter estimates for modified Beverton-Holt models of the abundances of juvenile emigrants expressing four alternative life-history strategies as a function of female spawner abundance. *α, γ,* and *J*^max^ are the parameters of the modified Beverton-Holt model, *lcl* represents the lower 95% confidence limit and *ucl* is the upper 95% confidence limit. *Spr-0* = spring subyearling, *Sum-0* = summer subyearling, *Fall-0* = fall subyearling, and *Spr-1* = spring yearling emigrants.

| stream | LH |  | *α* | *α* lcl | *α* ucl |  | *γ* | *γ* lcl | *γ* ucl |  | *J*^max^ | *J*^max^ lcl | *J*^max^ ucl |
| --- | --- | --- | --- | --- | --- | --- | --- | --- | --- | --- | --- | --- | --- |
| Chiwawa | Spr-0 |  | 4.5 | 1.7 | 11.6 |  | 1.84 | 1.45 | 2.34 |  | 14884 | 1184 | 186493 |
| Nason | Spr-0 |  | 5.5 | 2.3 | 12.9 |  | 1.25 | 0.89 | 1.76 |  | 14769 | 1147 | 189665 |
| White | Spr-0 |  | 5.6 | 3.0 | 10.7 |  | 1.59 | 1.07 | 2.36 |  | 14808 | 1153 | 187409 |
| Chiwawa | Sum-0 |  | 45.7 | 30.3 | 69.0 |  | 1.39 | 1.19 | 1.62 |  | 19743 | 2589 | 150045 |
| Nason | Sum-0 |  | 51.6 | 23.2 | 114.9 |  | 1.04 | 0.75 | 1.46 |  | 19153 | 2294 | 161315 |
| White | Sum-0 |  | 28.2 | 18.2 | 43.7 |  | 0.91 | 0.59 | 1.41 |  | 19183 | 2286 | 159249 |
| Chiwawa | Fall-0 |  | 104.3 | 63.7 | 171.3 |  | 1.16 | 0.71 | 1.88 |  | 1325 | 640 | 2766 |
| Nason | Fall-0 |  | 101.7 | 47.3 | 218.1 |  | 1.2 | 0.69 | 2.08 |  | 1515 | 590 | 3910 |
| White | Fall-0 |  | 75.4 | 42.7 | 132.1 |  | 0.71 | 0.34 | 1.53 |  | 1365 | 479 | 3916 |
| Chiwawa | Spr-1 |  | 474.7 | 333.8 | 677.5 |  | 0.35 | 0.22 | 0.56 |  | 14901 | 1148 | 203007 |
| Nason | Spr-1 |  | 112.9 | 57.2 | 224.1 |  | 0.31 | 0.13 | 0.73 |  | 14771 | 1094 | 207672 |
| White | Spr-1 |  | 134.2 | 68.0 | 265.2 |  | 0.35 | 0.14 | 0.85 |  | 14798 | 1111 | 203561 |


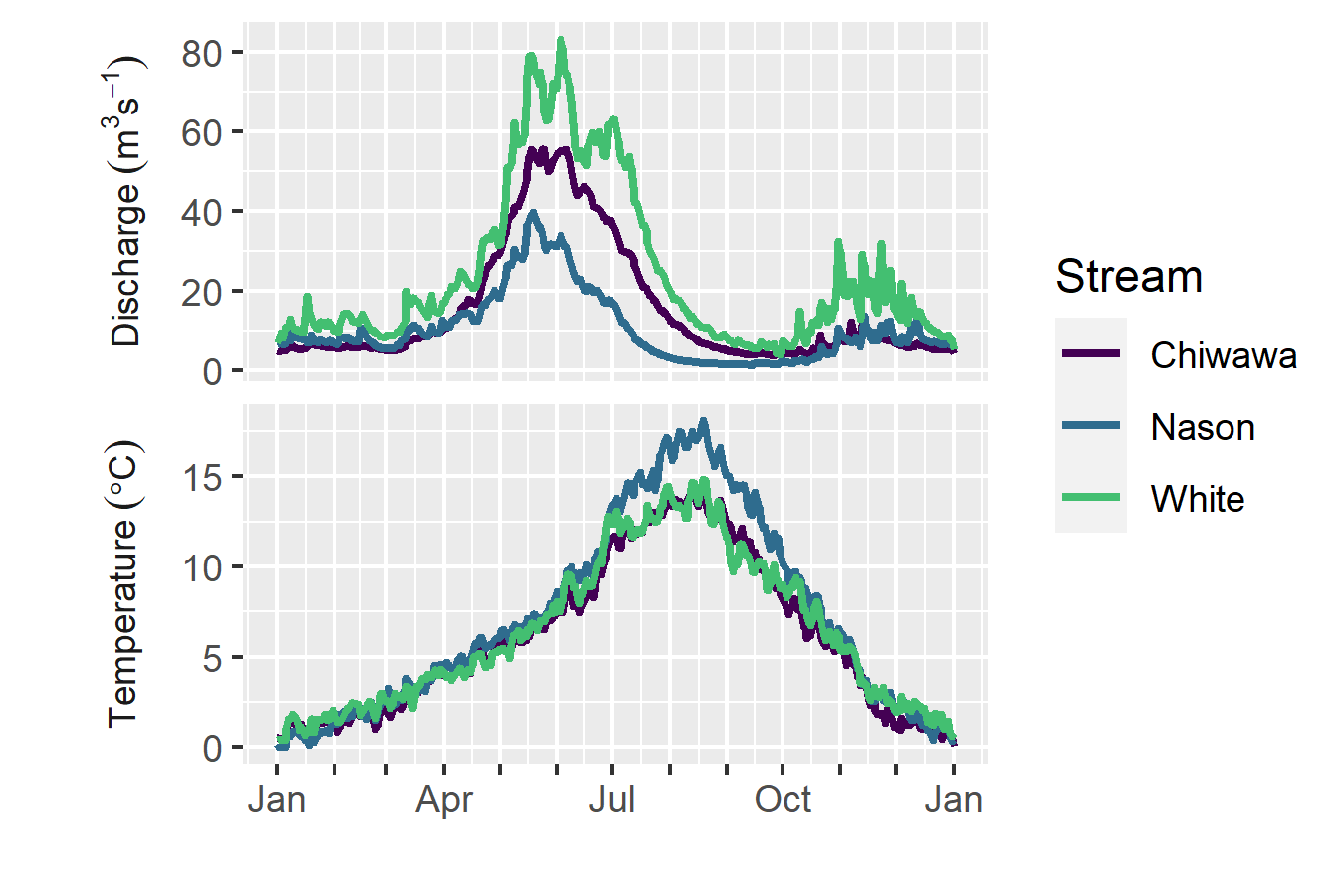


Figure S1. Average daily stream discharge (m^3^s^-1^) and temperature (°C) in the Chiwawa River, Nason Creek, and the White River in the Wenatchee River Basin. Discharge averages were taken over the years 1997–2018 in the Chiwawa, 2004–2018 in Nason Creek, and 2006–2018 in the White River. Temperature averages were taken over the years 2013–2017 in the Chiwawa River, 2012–2017 in Nason Creek, and 2014–2017 in the White River (Siegel and Volk 2019).


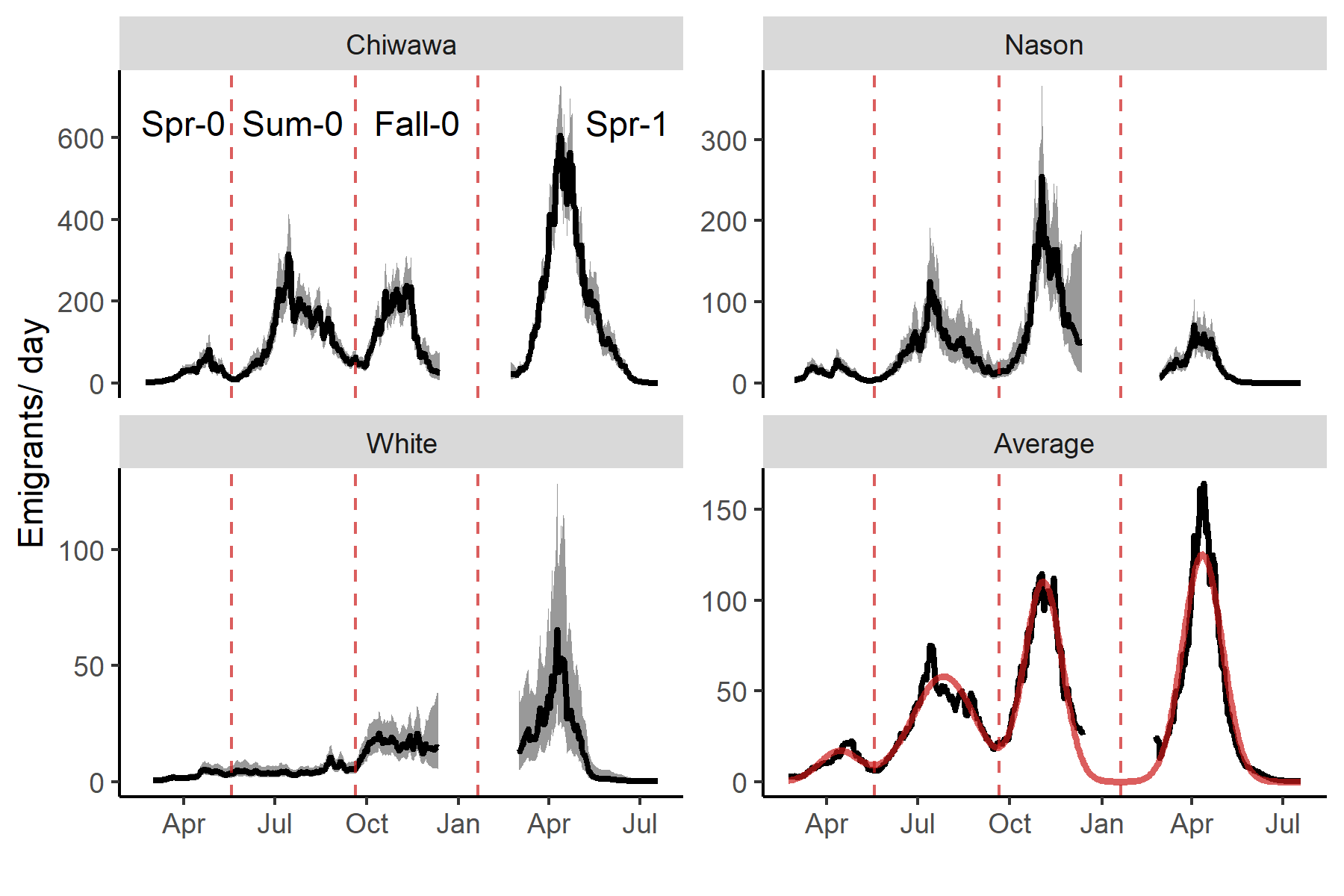


Figure S2. Mean daily numbers of emigrants across years in three natal streams. Gray shaded area is the 95% confidence interval. The red solid line is a mixture distribution used to identify breakpoints between juvenile life histories. The red dashed lines delineate the juvenile life-history pathways, defined by emigration day of year: *Spr-0* = spring subyearling, *Sum-0* = summer subyearling, *Fall-0* = fall subyearling, and *Spr-1* = spring yearling emigrants. The gap in outmigrant estimates is the period when no trapping was conducted due to ice in the river.

# References

Siegel, J. E., and C. J. Volk. 2019. Accurate spatiotemporal predictions of daily stream temperature from statistical models accounting for interactions between climate and landscape. PeerJ 7:e7892.
