# Supplementary material for: Effects of population density and environmental conditions on life‐history prevalence in a migratory fish
Source: Ecol Evol. 2023 May 23;13(5):e10087. doi: 10.1002/ece3.10087 (PMC10206029; doi:10.1002/ece3.10087)
Supplement: Supplementary file 2 — Appendix S2. [file ECE3-13-e10087-s002.docx]

**Appendix S2**

**Effects of population density and environmental conditions on life history expression in a migratory fish**

Mark H. Sorel^1^, Andrew R. Murdoch^2^, Richard W. Zabel^3^, Cory M. Kamphaus^4^, Eric R. Buhle^3,5^, Mark D. Scheuerell^6^, and Sarah J. Converse^7^

This appendix documents how we assigned ages to juvenile Chinook captured in screw traps in the Wenatchee River Basin based on their length and day of year of capture. Emigration form natal streams occured in three pulses each year (Figure S1), and the spring pulse included both age-0 and age-1 fish, which had different lengths (Figure S2).

A subset of emigrants >60 mm was implanted with passive integrated transponder tags, and a subset of those fish were detected when passing dams in the seaward-migration corridor. Based on the date of emigration and subsequent detection at downstream dams, we can infer the age that a fish was when it emigrated (Figure S2), because fish that emigrate at age 1 continue to the ocean in the same year and fish that emigrate at age 0 remain in the Wenatchee River Basin until the following year (Buchanan et al. 2015).

As a first step in developing a rule for classifying fish ages based on length and day of year at emigration, we determine the day of year before which 99.9% of fish that were known to have been age 1 at emigration were captured (Figure S3). The 99.9th quantile of capture days for 12,897 fish that were known to have been age 1 at emigration because they were subsequently detected in the migration corridor in the same year as emigration, was day 179. We therefore classified fish captured after day 179 as age 0.

The next step was to delineate age 0 and age 1 fish that emigration prior to day 179 based on their capture length and day of year. We used a three-step process to accomplish this task. First, we fit a mixture distribution of two normal distributions to the log-transformed lengths of all fish that had been captured within 10-day intervals (combining multiple years) starting on day 50 and ending on day 179 using the package *mixtools* in the R statistical environment (Benaglia et al. 2009; R Core Team 2021). Next, we found the length corresponding to the minimum densities between the two modes of the mixture distribution (Figure S4). Finally, we linearly interpolated between points represented by the midpoint of each 10-day interval and the length corresponding with the minimum density of the mixture distribution for that interval (Figure S2). Using the resulting line to delineate ages, this method correctly classified 99.91% of the 19,517 fish whose ages could be determined based on their date of capture and subsequent detection at dams in the migration corridor.

**References**

Benaglia T, Chauveau D, Hunter DR, Young D. 2009. mixtools: An R package for analyzing finite mixture models. Journal of Statistical Software **32**:1–29.

Buchanan RA, Skalski JR, Mackey G, Snow C, Murdoch AR. 2015. Estimating cohort survival through tributaries for salmonid populations with variable ages at migration. North American Journal of Fisheries Management **35**:958–973.

R Core Team. 2021. R: a language and environment for statistical computing. R Foundation for Statistical Computing, Vienna, Austria. Available from https://www.R-project.org/.


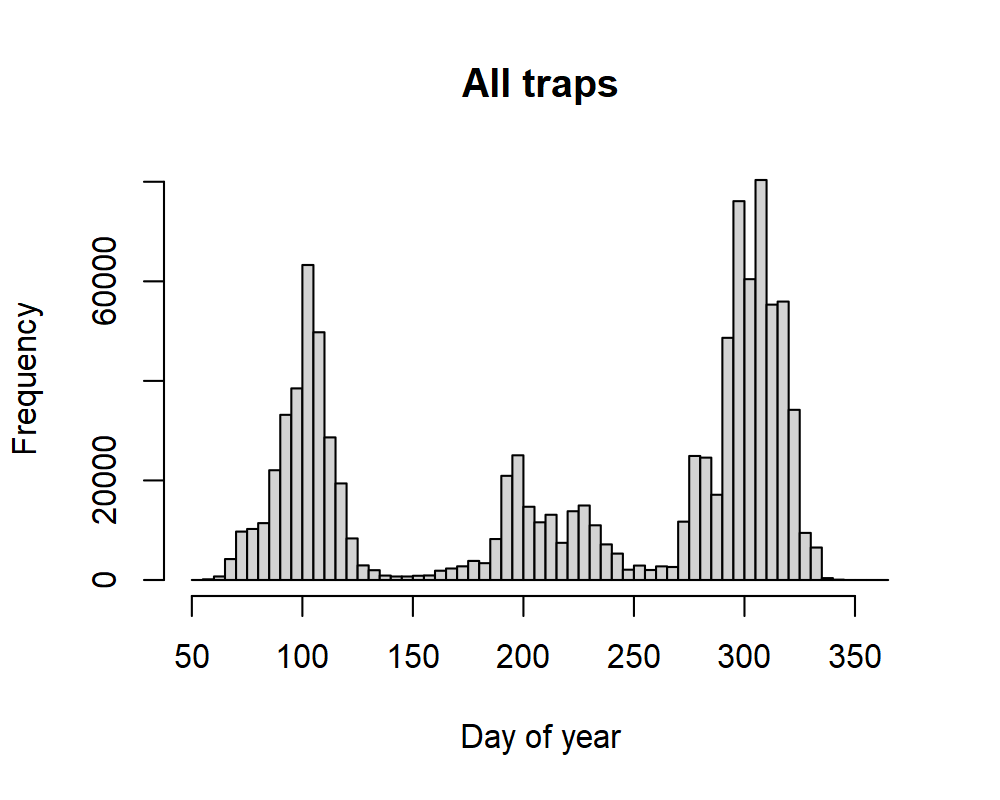


Figure S1. Histogram of the number of fish caught by day of year in three natal streams over multiple years.


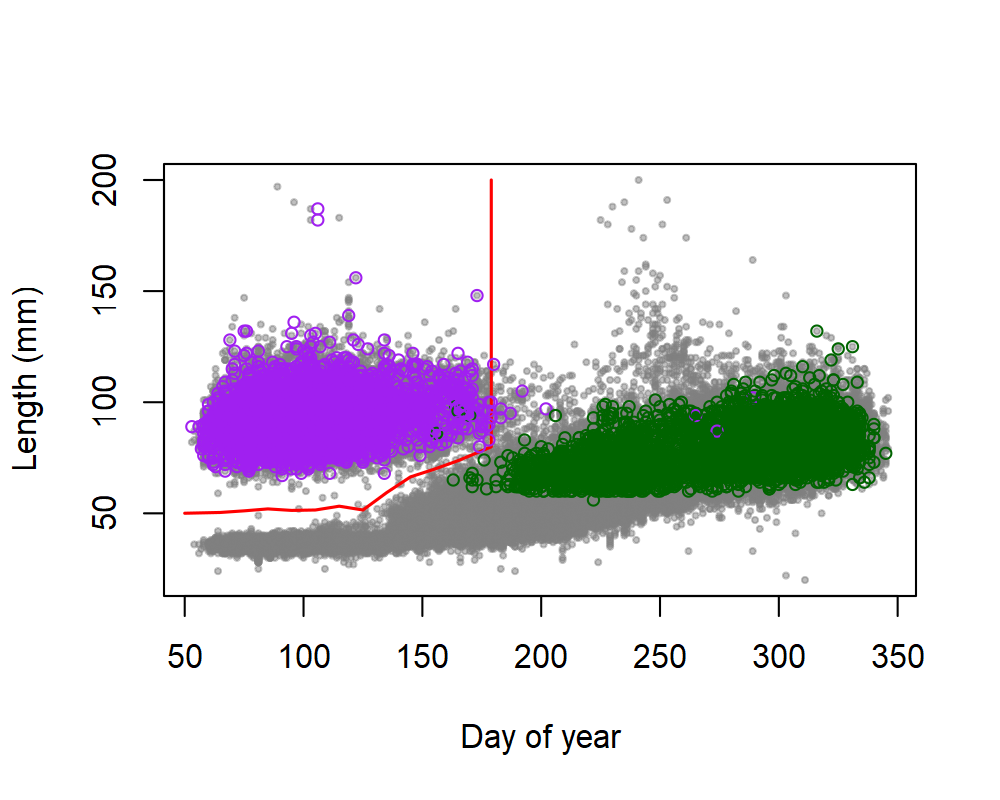


Figure S2. Cutoff line (red) for delineating age 1 (above line) from age 0 (below line) fish a based on their length and day of year of capture during emigration from natal streams. Grey points represent observed lengths and days of year of captured fish, purple points represent fish that were presumed to be age 1 at emigration based on the timing of their initial capture and subsequent detection in the migration corridor, and green points represent fish presumed to have been age 0 at emigration based on this information.


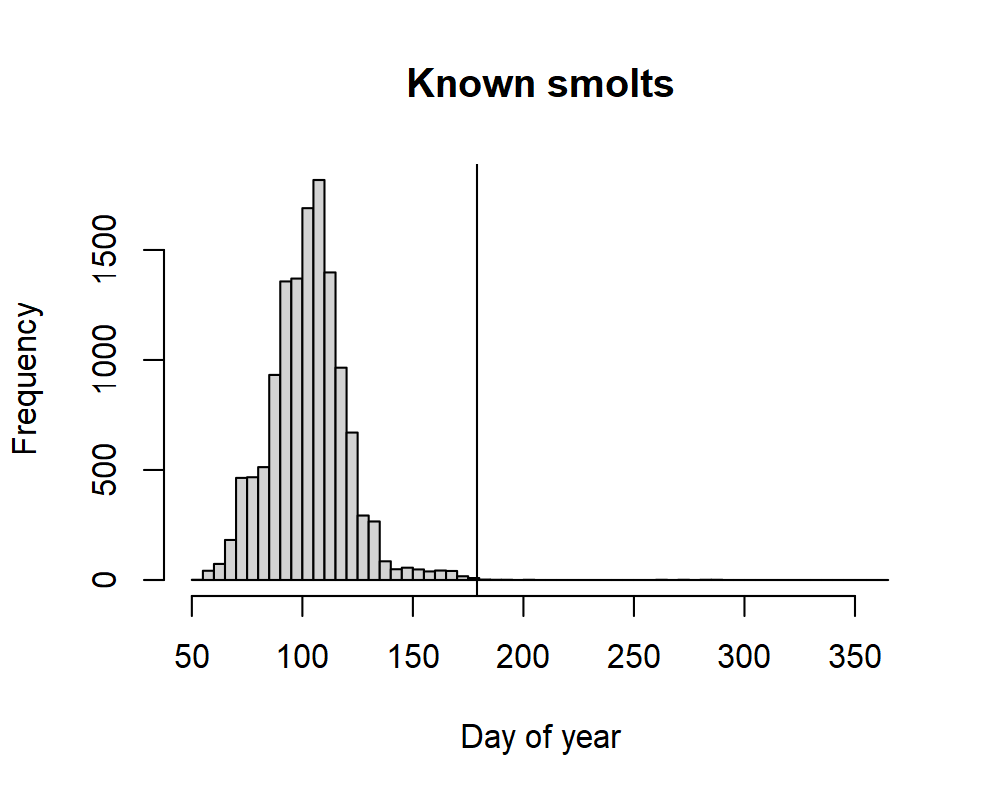


Figure S3. Histogram of capture day of year of fish that were tagged at screw traps and subsequently detected at mainstem dams when migrating downstream in the same year they were tagged. The vertical line represents the 99.9^th^ quantile of capture day of year for these fish.


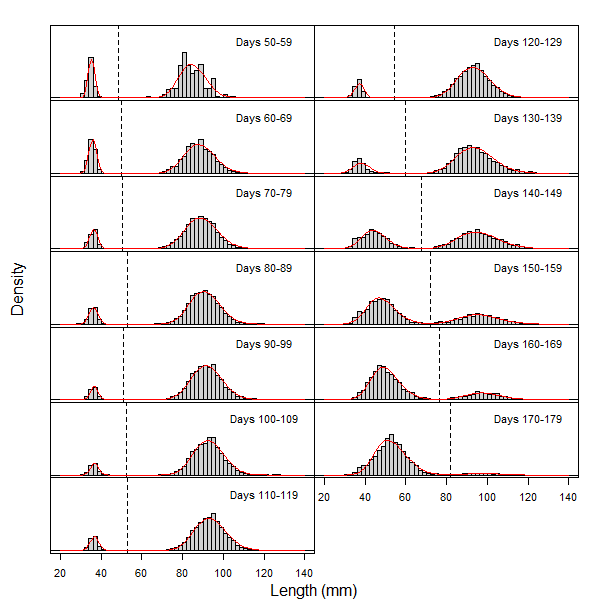


Figure S4. Histograms of the lengths of fish captured within 10-day intervals overlain with two-lognormal mixture distributions fit to these data (red line). The dashed vertical lines represent the minimum density of the mixture distribution between the two modes.
